# A Predictive Risk Score to Diagnose Adrenal Insufficiency in Outpatients: A 7 Year Retrospective Cohort Study

**DOI:** 10.3390/medicines8030013

**Published:** 2021-03-10

**Authors:** Worapaka Manosroi, Tanyong Pipanmekaporn, Jiraporn Khorana, Pichitchai Atthakomol, Mattabhorn Phimphilai

**Affiliations:** 1 Division of Endocrinology, Department of Internal Medicine, Faculty of Medicine, Chiang Mai University, Chiang Mai 50200, Thailand; worapaka.m@gmail.com; 2 Clinical Epidemiology Center, Faculty of Medicine, Chiang Mai University, Chiang Mai 50200, Thailand; tanyong24@gmail.com (T.P.); jiraporn.kho@elearning.cmu.ac.th (J.K.); 3 Clinical Statistic Center, Faculty of Medicine, Chiang Mai University, Chiang Mai 50200, Thailand; 4 Department of Anesthesiology, Faculty of Medicine, Chiang Mai University, Chiang Mai 50200, Thailand; 5 Division of Pediatric Surgery, Department of Surgery, Faculty of Medicine, Chiang Mai University, Chiang Mai 50200, Thailand; 6 Department of Orthopaedics, Faculty of Medicine, Chiang Mai University, Chiang Mai 50200, Thailand; p.atthakomol@gmail.com

**Keywords:** adrenal insufficiency, predictive model, serum cortisol, ACTH stimulation test

## Abstract

**Background:** The diagnosis of adrenal insufficiency (AI) requires dynamic tests which may not be available in some institutions. This study aimed to develop a predictive risk score to help diagnose AI in outpatients with indeterminate serum cortisol levels. **Methods:** Five hundred and seven patients with intermediate serum cortisol levels (3–17.9 µg/dL) who had undergone ACTH (adrenocorticotropin) stimulation tests were included in the study. A predictive risk score was created using significant predictive factors identified by multivariable analysis using Poisson regression clustered by ACTH dose. **Results:** The seven predictive factors used in the development of a predictive model with their assigned scores are as follows: chronic kidney disease (9.0), Cushingoid appearance in exogenous steroid use (12.0), nausea and/or vomiting (6.0), fatigue (2.0), basal cortisol <9 µg/dL (12.5), cholesterol <150 mg/dL (2.5) and sodium <135 mEq/L (1.0). Predictive risk scores range from 0–50.0. A high risk level (scores of 19.5–50.0) indicates a higher possibility of having AI (positive likelihood ratio (LR+) = 11.75), while a low risk level (scores of <19.0) indicates a lower chance of having AI (LR+ = 0.09). The predictive performance of the scoring system was 0.82 based on the area under the curve. **Conclusions:** This predictive risk score can help to determine the probability of AI and can be used as a guide to determine which patients need treatment for AI and which require dynamic tests to confirm AI.

## 1. Introduction

Adrenal insufficiency (AI) can be categorized into primary and secondary AI. The main causes of primary AI worldwide are tuberculosis and autoimmune adrenalitis [1,2]. Post-glucocorticoid therapy-induced AI has often been cited as the most common cause of secondary AI. Previous studies have identified multiple clinical and biochemical factors associated with AI [3,4,5,6,7], including a history of cirrhosis, autoimmune diseases, hepatitis C, HIV infection, chronic kidney disease (CKD), fatigue, nausea and vomiting, symptoms of hypotension and glucocorticoid use, and Cushingoid appearance. Biochemical factors associated with AI include low basal cortisol, cholesterol, and sodium [7].

Different protocols for AI diagnosis have been used in various institutions. Those suspected of having AI may proceed directly to adrenocorticotropin (ACTH) stimulation testing without screening for serum morning cortisol levels [8]. Some protocols propose that if serum cortisol is drawn at 08:00 and the level is below 3–5 µg/dL (83–138 nmol/L), it is strongly suggestive of AI and indicates that other dynamic tests, e.g., an ACTH stimulation test or insulin-induced hypoglycemia, are not necessary [8,9]. Another study suggested that if the 08:00 serum cortisol level is >15 µg/d (414 nmol/L), a diagnosis of AI is less likely [10]. When the serum cortisol levels are in the indeterminate range, dynamic tests are mandatory.

A frequently encountered problem in health care centers is the lack of access to diagnostic procedures such as ACTH stimulation tests or insulin-induced hypoglycemia tests. The ACTH stimulation test is currently the preferred diagnostic test as it is both safe and reliable [8]. In some institutions, the ACTH utilized in the tests may be in short supply or unavailable. In situations where supplies are limited, ACTH diluted to a low dose (1–5 µg) may be employed instead of the usual high dose of ACTH (250 µg). The use of diluted ACTH can lead to errors in the interpretation of the results and diagnosis if the diluted ACTH is not properly prepared [8].

Multiple reports have recommended upper and lower cut-off levels for serum cortisol levels to help rule out and rule in the presence of AI [3,11]. It has been estimated that using these cut-off levels could potentially diminish the number of dynamic tests by approximately 30% [3]. However, a problem occurs when serum cortisol levels fall in the intermediate level where AI cannot be either excluded or diagnosed; in those cases, dynamic tests are mandatory. A simple predictive tool that incorporates readily available clinical and laboratory data and that increases the accuracy of the prediction of AI could potentially reduce the number of ACTH stimulation procedures. Such a method would be particularly valuable where supplies of ACTH are limited or nonexistent. To date, there have been no reports of such a tool that could predict the risk of AI in cases where serum cortisol levels are in the intermediate range.

This study aimed to design a simple-to-use predictive score based on easy-to-obtain clinical and biochemical parameters to facilitate the prediction of secondary AI in patients with intermediate levels of cortisol.

## 2. Materials and Methods

A 7 year retrospective cohort study was conducted at the adult endocrinology outpatient department unit of Maharaj Nakhon Chiang Mai Hospital, Thailand. All data were acquired during January 2010–December 2016. The study was approved by the institutional board review of the Faculty of Medicine, Chiang Mai University. The ethical code is EXEMPTION-6193/2019 and date of approval is 27 March 2019. Informed consent was waived by the ethics committee. Inclusion criteria were adult patients aged more than 18 years with 08:00 serum morning cortisol between 3–17.9 µg/dL (83–500 nmol/L) who had undergone ACTH stimulation testing. We excluded the patients suspected of having primary AI or congenital adrenal hyperplasia, those with incomplete data for ACTH stimulation tests, females currently on hormonal therapy or taking oral contraceptive pills containing estrogen, and patients who had undergone pituitary surgery within the previous 2 months. The method used has been described in a previous study conducted by the authors [7].

### 2.1. ACTH Stimulation Test Protocol

Details of the ACTH stimulation test protocol have been described previously [7]. In brief, patients currently taking glucocorticoids are instructed to discontinue the medications at least 24 h before the tests. During May 2010–March 2014, only low-dose ACTH stimulation tests were performed in Thailand due to a shortage of ACTH. Serum cortisol was obtained at 0 (basal cortisol), 30, and 60 min after either 1 or 250 µg of ACTH had been administered intravenously.

### 2.2. Definitions

In this study, AI was defined as a peak serum cortisol level at 30 or 60 min after ACTH stimulation of less than 18 µg/dL (<500 nmol/L). Normal adrenal response was defined as a peak serum cortisol level 30 or 60 min after ACTH stimulation of ≥18 µg/dL (≥500 nmol/L). CKD was diagnosed if the patient had an estimated glomerular filtration rate (eGFR) of less than 30 mL/min/1.73 m^2^ as calculated using the modification of diet in renal disease (MDRD) formula. Fatigue, nausea/vomiting, and orthostatic hypotension were symptoms reported by the patients and documented in the medical record by a medical practitioner. The definition of weight loss was a loss of 5% of body weight in one month or 10% over a period of six months or longer [12]. Cushingoid appearance was defined as at least one sign of glucocorticoid excess documented in the medical record by the medical practitioners, e.g., moon face, facial plethora, dorsocervical fat pad, proximal muscle weakness, easy bruising, and hirsutism [7].

### 2.3. Predictive Variables

Clinical and biochemical data were obtained from electronic medical records. Clinical data included demographic information, e.g., age, sex, and underlying diseases. Indications for ACTH stimulation testing were also collected. Biochemical factors such as serum albumin, creatinine, and cholesterol were acquired within 3 months before or following the tests. Serum 08:00 morning cortisol and basal cortisol levels were obtained using an electrochemiluminescence immunoassay (ECLIA) (Elecsys^®^ Cortisol 1010, Roche Diagnostics, Laval, QC, Canada). The intra- and inter-assay coefficients of variation for serum cortisol were <10%.

### 2.4. Outcome Variable

The results of the ACTH stimulation tests were categorized into 2 groups: AI and normal adrenal response.

### 2.5. Statistical Analysis

STATA program version 15.1 (Stata Corp., College Station, TX, USA) was used for analysis. The statistical significance level was defined as p-value < 0.05 for two-tailed tests. Data are demonstrated either as count and percentage or as mean and standard deviation (SD). Fisher’s exact test and the *t-*test or the Mann–Whitney *U* test were performed for univariable comparative statistics for categorical and continuous data, respectively. Poisson regression clustered by ACTH dose was performed using multivariable analysis; the results are reported as a coefficient value and a 95% confidence interval (CI). Significant predictive factors identified in a multivariable model from an earlier study were employed in the current model to predict AI [7].

Item scores were calculated by the transformation of the regression coefficient. The coefficient of each level for each factor was divided by the smallest coefficient of the model and rounded to the nearest 0.5. Item scores were then added together to calculate a total score. The total scores were then divided into 2 risk levels: groups at a low risk and at a high risk of having AI. The cut-off point for the risk levels was acquired from the level which yielded the lowest positive likelihood ratio (LHR+) of AI and the highest LHR+ of AI for the low-risk and the high-risk group, respectively. Discrimination of the prediction scores is presented as the area under the receiver operating characteristic (AuROC) curve and a 95% CI. Internal validation was performed using a resampling technique (bootstrapping method) and the concordance index (C-index) was reported. To give 80% power at the 5% significance level (two-sided with an odds ratio of 0.42 of detecting AI for a specific risk factor), a sample size of at least 430 patients was estimated to be needed [3].

## 3. Results

A total of 527 patients who had serum morning cortisol between 3–17.9 μg/dL were included in this study. Three patients with serum morning cortisol <3 or ≥18 μ/dL, 2 patients with incomplete results from the ACTH stimulation tests, 1 patient who was on oral contraceptive pills, 2 patients who had pituitary surgery in the past 2 months, 2 patients with congenital adrenal hyperplasia, and 10 patients with primary AI were excluded. Therefore, 507 patients were enrolled. Baseline characteristics and biochemical investigation results are shown in Table 1. A total of 507 patients were included in the predictive model analysis. Of these, 24.7% (n = 125/507) were diagnosed with AI. AI was significantly more common in patients aged ≥50; those with hypertension, CKD, fatigue, or a history of pituitary surgery; and in patients with exogenous steroid use and Cushingoid appearance. Baseline biochemical investigations found that serum albumin was significantly lower in the AI group than in the normal adrenal response group (*p* < 0.001).

**Figure 1:**
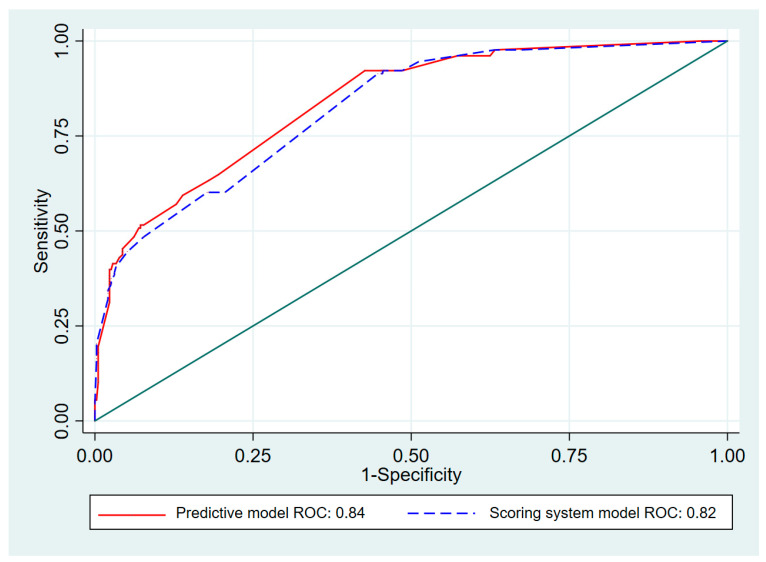
Area under receiver operating characteristic (AuROC) curve of adrenal insufficiency predicted by risk scoring system and predictive model (curved line) and 50% chance prediction (diagonal line).

Seven initial predictors of AI were chosen based on the predictive clinical factors previously reported by Manosroi et al. [7]. Those factors were CKD, Cushingoid appearance in patients with exogenous steroid use, symptoms of nausea/vomiting, and fatigue. The biochemical factors were serum basal cortisol <9 µg/dL (<248 nmol/L), serum cholesterol <150 mg/dL, and serum sodium <135 mEq/L. Risk scoring was created to predict the probability of patients with a normal adrenal response having AI. The transformed scores ranged from 1.0 to 13.5. The scoring scheme is shown in Table 2. The predictive ability of the scoring system with the transformed scores of all seven predictive factors represented by AuROC was 0.82, 95% CI (0.78–0.86), which is similar to the predictive ability of the model before transforming the scoring system (AuROC 0.84) (95% CI 0.80–0.88) (Figure 1).

**Figure 2:**
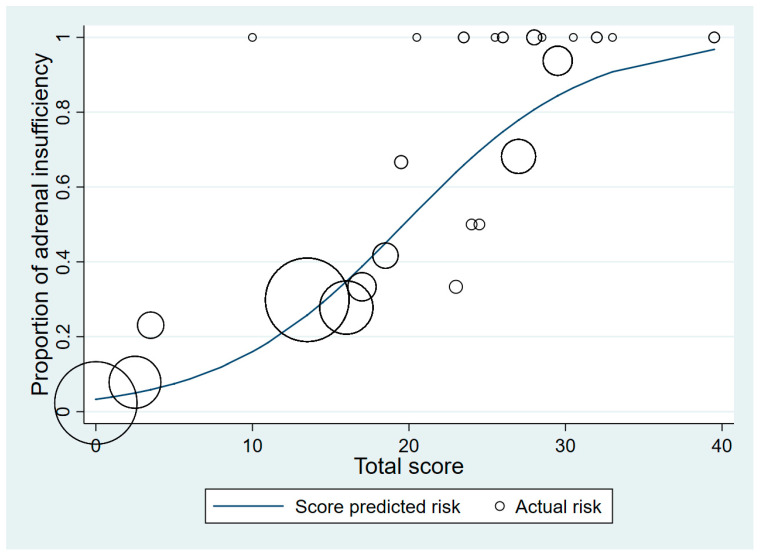
Score-predicted risk (line) and actual risk (circles) of adrenal insufficiency for each total score (the size of the circle indicates the number of patients).

The total scores were classified into two groups: a low-risk group (scores 0–20.0) and a high-risk group (scores 20.5–50.0) (Table 3 and Table 4). Patients with a normal adrenal response were more common in the low-risk group (72.9%). In the low-risk group, 370 patients had normal adrenal responses and 77 patients had AI, which demonstrated a 61.6% specificity. In the high-risk group, 48 patients had AI while 12 patients had normal adrenal responses, which showed a 96.9% specificity. Figure 2 shows the relationship between the proportion of patients with AI and the total scores. The higher the score, the greater the proportion with AI. The accuracy of our model was further verified by bootstrap validation. The C-index was 0.77 (95% CI 0.72–0.82). The proposed predictive criteria are shown in Table 4.

## 4. Discussion

The present study has proposed that the predictive risk score system for facilitating the prediction of AI shows good diagnostic accuracy: 82% based on AuROC. This clinical prediction model represents a simple and affordable tool to facilitate the diagnosis of AI. Present AI diagnostic procedures require multiple steps, including the screening for serum morning cortisol followed by ACTH stimulation tests. In institutions where ACTH is not available, patients suspected of having AI may need to be transferred to other institutions where the tests are available. With the predictive risk score system, the number of patient referrals as well as the number of tests could potentially be reduced, representing time and cost savings for patients, healthcare practitioners, and health care facilities.

To maximize the potential for high diagnostic specificity (96.9%) and to minimize the false positive rate, a high LHR+ for the cut-off point was employed for the group with a high risk of having AI. Similarly, a low LHR+ cut-off point was used with the group at low risk of having AI in order to minimize the number of false negatives. A single cut-off point that demonstrates both high sensitivity and high specificity simultaneously cannot be achieved. To reduce the number of false positive diagnoses, the proposed cut-off demonstrated a high specificity for diagnosing AI. The scoring system categorized patients into two groups: those with a high risk of AI and those with a low risk. Patients with scores above 20.5 were in the high-risk group. Cushingoid appearance in patients with exogenous glucocorticoid use and those with serum basal cortisol levels <9 µg/dL (<248 nmol/L) who scored 12.0 and 12.5 each, respectively, played a major role in the predictive score. Patients who had at least one of these factors in addition to other factors were promptly categorized into the AI high-risk group. In terms of clinical application, easy-to-use predictive criteria for AI were suggested based on the risk score system.

Of the patients at high risk for AI based on their predictive risk score, all but 12 had AI. Based on these findings, we recommended that those in the high-risk group proceed directly to AI treatment including the initiation of physiologic doses of glucocorticoid. For those in the low-risk group, we recommended dynamic tests such as the ACTH stimulation test to rule out AI and to preclude a misdiagnosis of this disease, as a misdiagnosis of AI can potentially lead to a critical and even life-threatening situation. Applying this predictive risk score system to patients suspected of having AI could potentially decrease the number of ACTH stimulation tests by 9.5% (n = 48/507). This clinical prediction model is intended for use with patients who have intermediate serum morning cortisol levels, i.e., levels between 3–17.9 µg/dL (83–500 nmol/L). It can help to guide decision making by physicians regarding whether or not further dynamic tests are indicated.

The proposed predictive risk score incorporates both clinical and biochemical predictive factors. Some of the variables in the final clinical prediction model have been stated in previous studies to be related with AI, including Cushingoid appearance in exogenous steroid use patients, nausea/vomiting, fatigue, low basal cortisol, low serum cholesterol, and hyponatremia [5,7,13,14,15,16]. Among the factors included in the model, Cushingoid appearance and serum basal cortisol levels <9 µg/dL showed a very high level of association with AI. Data regarding the association between AI and basal cortisol levels were recently published by our group [11]. That report showed that basal cortisol can be employed as an alternative method for the diagnosis of AI. The relationship between CKD and AI remains controversial, with some studies reporting that most CKD patients have normal adrenal function, while another study indicates that higher serum creatinine is associated with a lower risk of having AI [17,18,19].

To the best of our knowledge, previously reported tools to help diagnose AI have all been evaluated exclusively in cirrhotic patients, including the screening and diagnostic algorithms [20]. Other studies have explored groups of factors which can potentially predict the occurrence of AI [3,21]. The present study developed a simple and practical scoring system with good diagnostic accuracy that is suitable for use in normal clinical practice. A strength of this study is that the population used to create the scoring system had various indications of ACTH stimulation testing. Thus, this led to an advantage in terms of the generalizability of the new scoring system. Additionally, the present study had a large sample size, which provided adequate power of analysis. Finally, the observed relationships are not likely to have occurred by chance, as most of the factors related to AI in this study can be explained by the underpinning pathophysiology.

We acknowledge some limitations in this study. Symptoms of nausea/vomiting and fatigue are subjective and are only perceived by the patient; the documentation of these symptoms depends on the decision of the clinicians. Additionally, only patients with intermediate serum cortisol levels (between 3–17.9 µg/dL (83–500 nmol/L)) were included, making the results most relevant to that subgroup. Although the summary of recommendations from the Endocrine Society guidelines suggested that the low-dose (1 μg) corticotropin test for the diagnosis of AI can be used when the substance is in short supply [8]. The variation within and between low-dose synacthen dilution methods can provide an inaccurate dosage, leading to invalid results [22].

Finally, this study was internally verified based on a patient population in a single institution; external validation should be accomplished to confirm the predictive ability of this model.

## 5. Conclusions

The proposed predictive risk score system and criteria to diagnose secondary AI has an acceptable diagnostic accuracy. This system can potentially reduce the number of dynamic ACTH stimulation tests required, saving time, money, and resources. The scoring system can be utilized as a guide for clinicians in institutions where ACTH stimulation testing is limited or not available. In low-risk groups (scores 0–20.0), ACTH stimulation tests or other dynamic tests should be required. In high-risk groups (scores 20.5–50.0), AI treatment is indicated. Future external validation of this predictive risk score is warranted.

## Figures and Tables

**Table 1:** Baseline characteristics and biochemical investigation results of patients with adrenal insufficiency (n = 125) and normal adrenal response (n = 382).

Characteristic	Adrenal Insufficiency (n = 125)	Normal Adrenal Response (n = 382)	*p*-Value
**Baseline Characteristics**			
Age			
- <50 years, n (%)	40 (32.0)	170 (45.5)	
- ≥50 years, n (%)	85 (68.0)	212 (55.5)	0.016 *
Male, n (%)	67 (53.6)	194 (50.8)	0.607
ACTH stimulation dose, n (%)			
- 1 µg	41 (32.8)	148 (38.7)	
- 250 µg	87 (67.2)	234 (61.3)	0.243
Underlying disease, n (%)			
- Diabetes mellitus	19 (15.2)	56 (14.7)	0.885
- Hypertension	40 (32.0)	83 (21.8)	0.023 *
- Chronic kidney disease	11 (8.8)	5 (1.3)	<0.001 *
- Autoimmune disease	24 (19.2)	52 (13.6)	0.149
- Cancer	3 (2.4)	12 (3.1)	0.671
Symptom, n (%)			
- Fatigue	36 (28.8)	70 (18.3)	0.016 *
- Weight loss	4 (3.2)	22 (5.8)	0.352
- Orthostatic hypotension	14 (11.2)	36 (9.4)	0.604
- Nausea/vomiting	4 (3.2)	5 (1.3)	0.234
Indication for ACTH testing, n (%)			
- Exogenous steroid use	57 (45.6)	73 (19.1)	<0.001 *
- Post-surgery of pituitary	18 (14.4)	85 (22.3)	0.072
- Pituitary tumor	20 (16.0)	94 (24.6)	0.049 *
- Pituitary hormonal deficiencies	32 (25.6)	115 (30.1)	0.365
- Symptoms of adrenal insufficiency	40 (32.0)	130 (34.0)	0.744
- Hyponatremia	10 (8.0)	8 (2.1)	0.004 *
- Hypoglycemia	2 (1.6)	12 (3.1)	0.534
Cushingoid appearance in exogenous steroid use	49 (39.2)	15 (3.9)	<0.001 *
History of pituitary surgery, n (%)			
- Microadenoma	6 (17.6)	31 (18.4)	
- Macroadenoma	28 (82.4)	137 (81.6)	0.912
Other hormonal deficiencies, n (%)			
- Gonadotropin	10 (12.8)	45 (20.9)	0.148
- Thyroid	26 (32.1)	77 (32.9)	0.894
- Growth hormone	4 (5.4)	13 (6.7)	0.787
- Diabetes insipidus	5 (6.9)	28 (13.9)	0.143
**Baseline biochemical investigations**			
Serum morning cortisol (µg/dL)			
- <9 µg/dL, n (%)	81 (64.8)	184 (48.2)	
- ≥9 µg/dL, n (%)	44 (35.2)	198 (51.8)	0.001 *
Serum basal cortisol (µg/dL)			
- <9 µg/dL, n (%)	101 (80.8)	158 (41.4)	
- ≥9 µg/dL, n (%)	24 (19.2)	224 (58.6)	<0.001*
Serum albumin (g/dL)			
- <3 g/dL	20 (16.0)	16 (4.2)	
- ≥3 g/dL	105 (84.0)	366 (95.8)	<0.001 *
Total cholesterol (mg/dL)			
- <150 mg/dL	38 (30.4)	79 (20.7)	
- ≥150 mg/dL	87 (69.6)	303 (79.3)	0.028 *
Serum sodium (mEq/L)			
- <135 mEq/L	19 (15.2)	54 (14.1)	
- ≥135 mEq/L	106 (84.8)	328 (85.9)	0.770

* Statistical significance.

**Table 2:** Multivariate analysis with coefficient values of each factor.

Predictive Factors	RR	95% CI for RR	Coefficient	Transformed Coefficients	Assigned Score	*p*-Value
Chronic kidney disease						
- No			-			
- Yes	2.5	2.02–3.10	0.92	9.17	9	<0.001
Cushingoid appearance in exogenous steroid use						
- No						
- Yes			-			
	3.38	2.10–5.44	1.22	12.17	12	<0.001
Nausea and/or vomiting						
- No			-			
- Yes	1.82	1.20–2.76	0.6	6	6	0.005
Fatigue						
- No			-			
- Yes	1.24	1.14–1.35	0.22	2.15	2	<0.001
Basal cortisol						
- ≥9 µg/dL			-			
- <9 µg/dL	3.4	3.28–3.53	1.22	12.25	12.5	<0.001
Cholesterol						
- ≥150 mg/dL			-			
- <150 mg/dL	1.28	1.26–1.30	0.25	2.5	2.5	<0.001
Sodium						
- ≥135 mEq/L			-			
- <135 mEq/L	1.11	1.04–1.19	0.1	1	1	0.003

RR: Risk ratio.

**Table 3:** Risk score for the diagnosis of adrenal insufficiency.

Criteria	Score
Basal cortisol < 9 µg/dL	
Yes	12.5
No	0
Cushingoid appearance in exogenous steroid use	
Yes	12
No	0
Chronic kidney disease	
Yes	9
No	0
Nausea and/or vomiting	
Yes	6
No	0
Cholesterol < 150 mg/dL	
Yes	2.5
No	0
Fatigue	
Yes	2
No	0
Sodium < 135 mEq/L	
Yes	1
No	0
High risk of adrenal insufficiency if the total score is >20.5	

**Table 4:** Distribution of risk of adrenal insufficiency, LR+ and 95% CI of LR+.

Risk Level	Score	Adrenal Insufficiency (n = 125), n (%)	Normal Adrenal Response (n = 382), n (%)	LR+	95% CI of LR+	*p*-Value	Specificity (%)	Sensitivity (%)
Low	0–20.0	77 (15.2)	370 (72.9)	0.08	0.04–0.15	<0.001	61.6	3.1
High	20.5–50.0	48 (9.5)	12 (2.4)	12.22	6.71–22.26	<0.001	96.9	38.4

LR+: positive likelihood ratio.

## Data Availability

The data presented in this study are available on request from the corresponding author.
